# Supplementary figures and images for: Feasibility and Efficacy of Low-to-Moderate Intensity Aerobic Exercise Training in Reducing Resting Blood Pressure in Sedentary Older Saudis with Hypertension Living in Social Home Care: A Pilot Randomized Controlled Trial
Source: Medicina (Kaunas). 2023 Jun 18;59(6):1171. doi: 10.3390/medicina59061171 (PMC10301309; doi:10.3390/medicina59061171)

### CONSORT 2010 Flow Diagram

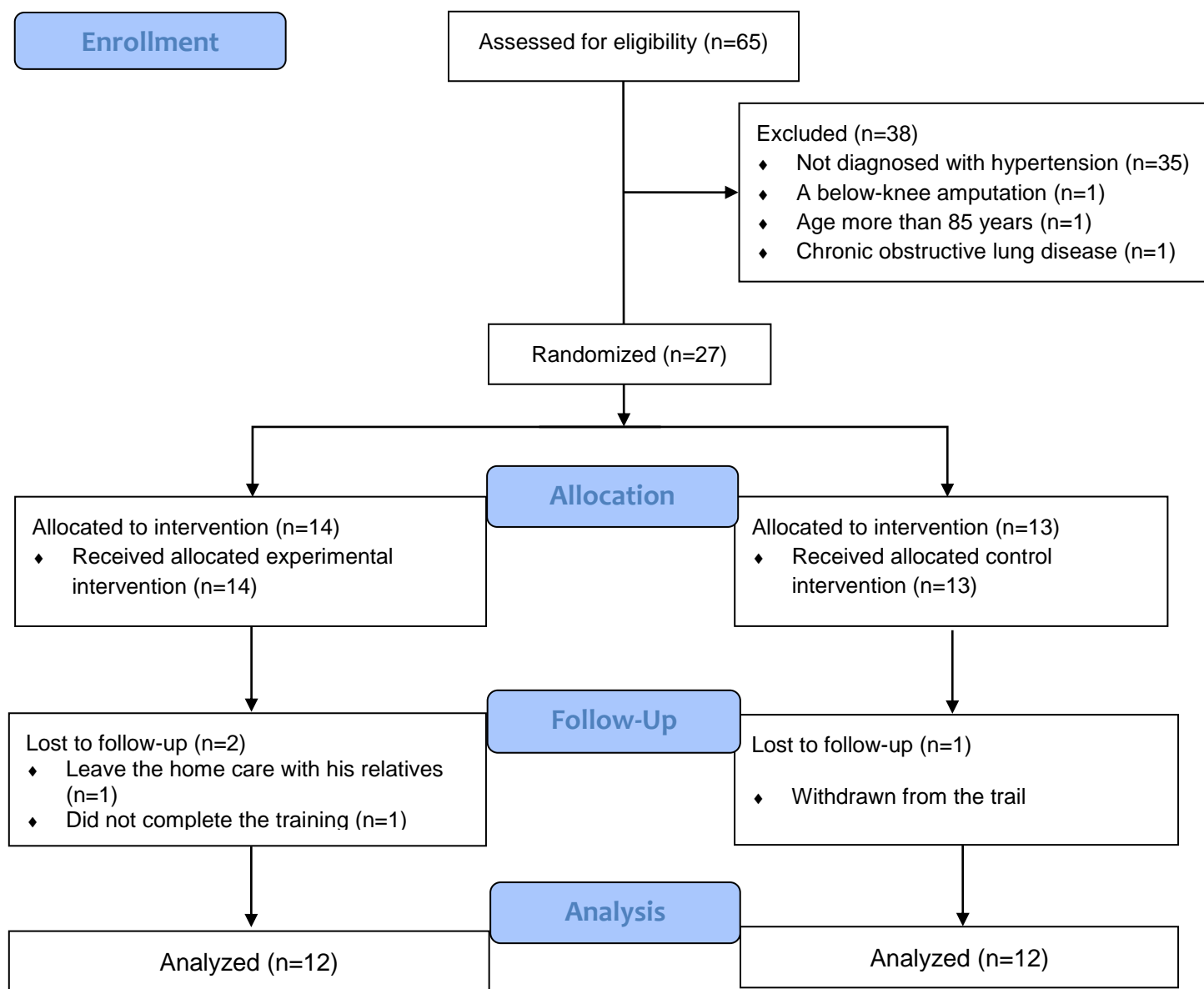

**Figure S1:** CONSORT 2010 flow diagram.

Supplement: Supplementary file 1 [file medicina-59-01171-s001.zip › medicina-2403913-supplementary.pdf]
